# Intelligent diagnosis of ovarian cancer in PET/CT imaging based on KiteNet-MobileNetv3 fusion and CarveMix augmentation

**DOI:** 10.1038/s41598-026-47682-5

**Published:** 2026-04-14

**Authors:** Junwei Li, Kai Hu, Xueru Fan, Sisheng Wang, Tao He, Caiyun Xu, Qiwen Cai, Jinyan Chen, Yating Ling, Jianmin Yang, Xiaohui Fan, Dexing Kong, Lixia Zhang, Lu Li

**Affiliations:** 1 Pharmaceutical Informatics Institute, School of Pharmacy and Women’s Hospital, School of Medicine, Zhejiang University, #866 Yuhangtang Road, Hangzhou, 310058 China; 2 State Key Laboratory of Chinese Medicine Modernization, Innovation Center of Yangtze River Delta, Zhejiang University, Jiaxing, 314102 China; 3 School of Mathematical Sciences, Zhejiang University, #866 Yuhangtang Road, Hangzhou, 310027 China; 4 Nuclear Medicine Department, The First Affiliated Hospital of Zhejiang Chinese Medical University (Zhejiang Provincial Hospital of Chinese Medicine), Hangzhou, 310006 China; 5 Sport Hospital of Zhejiang College of Sports, Hangzhou, 311231 China; 6 Department of Obstetrics and Gynaecology, Li Ka Shing Institute of Health Sciences, School of Biomedical Sciences, Sichuan University-Chinese University of Hong Kong Joint Reproductive Medicine Laboratory, The Chinese University of Hong Kong, Shatin, N.T., Hong Kong China; 7 Modern Chinese Medicine and Reproductive Health Joint Innovation Center and International Science and Technology Cooperation Base of Chinese Medicine Modernization and Big Health, Innovation Center of Yangtze River Delta, Zhejiang University, Jiaxing, 314102 China

**Keywords:** Ovarian cancer, PET/CT, Deep learning, Convolutional neural network, Intelligent diagnosis, Ovarian cancer, Cancer imaging

## Abstract

Ovarian cancer refers to a malignant tumor that grows in the ovary, which has the highest mortality rate of gynecological cancers. Positron emission tomography and computer tomography (PET/CT) imaging is widely used for the localization and characterization of ovarian tumors, but its analysis is susceptible to the subjectivity of clinicians. A deep learning-based PET/CT image diagnosis approach was investigated to achieve the segmentation and classification of ovarian tumors. We proposed several traditional convolutional neural networks (CNNs) for automatic ovarian tumors segmentation and classification. For segmentation, we design a hybrid network that integrates U-Net-MobileNetv3 with KiteNet to jointly capture global structure and lesion edge details, further enhanced by lesion-aware CarveMix augmentation and Dice-CE loss. For classification, we adopt ConvNeXt as the backbone and improve its robustness via Mixup data augmentation. Our method achieves a Dice coefficient of 0.826 and an accuracy of 0.912, outperforming all baseline models including U-Net, Deeplabv3, DenseNet, and Swin-Transformer. 1228 PET/CT images were employed to train and evaluate the CNN approach. Our segmentation model obtained a Dice of 0.826 compared to 0.773 obtained by U-Net-MobileNetv3, 0.444 by KiteNet, 0.634 by FCN, 0.744 by Deeplabv3, 0.667 by U-Net, and 0.747 by U-Net-VGG. Our classification model achieved the ACC of 0.912 compared to 0.907 achieved by ConvNeXt, 0.898 by DenseNet, 0.895 by EfficientNet and 0.848 by Swin-Transformer. In this study, we developed a novel deep learning framework for the simultaneous segmentation and classification of ovarian tumors in PET/CT imaging. Our key findings are: (1) A hybrid segmentation architecture that integrates U-Net-MobileNetv3 with KiteNet, enhanced by lesion-aware CarveMix augmentation and Dice-CE loss, achieved a Dice coefficient of 0.826, significantly outperforming standard models such as U-Net (0.667) and Deeplabv3 (0.744); (2) A classification pipeline based on ConvNeXt with Mixup data augmentation attained an accuracy of 0.912, surpassing DenseNet (0.898) and Swin-Transformer (0.848). These results demonstrate that our method can effectively support clinicians in both precise tumor delineation and reliable benign/malignant differentiation, offering a promising tool for intelligent ovarian cancer diagnosis.

## Introduction

Ovarian cancer is a malignant tumor that grows in the area of a woman’s ovaries^1^. Due to the lack of symptoms in the early stage of ovarian cancer and the limited effectiveness of screening, it is difficult to diagnose early^2^. At the time of the initial visit, 70% of patients are in an advanced stage, and the five-year survival rate of conventional surgery combined with postoperative adjuvant chemoradiotherapy is only 25–30%^3^. Therefore, it is the most common cause of death of all gynecologic cancers^4^. Over the past decade, scientific research has focused on improving ovarian cancer outcomes through the use of imaging techniques and serum markers to screen for preclinical and early-stage diseases^5–7^. Positron emission tomography and computer tomography (PET/CT) are important in the localization and characterization of ovarian tumors^8^. ^18^F-FDG PET/CT shows higher sensitivity, specificity, and accuracy (ACC) than traditional morphological diagnosis by increased tumor glucose metabolism and has a higher negative predictive value, which can avoid some unnecessary invasive tests and save medical costs^9^. Although PET/CT is important for the prognosis of malignancy, artificially interpreted imaging is inefficient and susceptible to clinician subjectivity. The application of deep learning algorithms in multimodal medical image-assisted diagnosis systems has many advantages^10^ and is expected to solve the problem of insufficient traditional shallow machine learning capabilities, thereby greatly improving auxiliary diagnosis capabilities.

Convolutional neural networks (CNNs) are successful image-processing models proposed recently^11^. Regarding the processing of ovarian ultrasound images, the existing research on early diagnosis algorithms mainly focused on extracting artificially designed features from ovarian ultrasound images and then supervising and classifying the extracted features through current machine learning classifiers^12–17^. This limits the use of medical diagnostic procedures based on computer-aided diagnostics. Mikko et al. distinguished healthy and ovarian cancer tissues from mice based on the second harmonic generation and two-photon excitation fluorescence contrast of multiphoton microscopy^18^. Moreover, Wang et al. used MR images to distinguish between ovarian junctional tumors and epithelial cancers based on a deep learning model, with an ACC rate of only 83.7%^19^. Dastidar et al.^20^ utilized the IARD algorithm for ovarian tumor segmentation on MRI images. They found that the algorithm was useful for measuring ovarian tumors in patients without significant ascites but failed to achieve the same results in patients with them. Hu et al.^21^ assessed the feasibility of deep learning methods in automatically segmenting epithelial ovarian cancer on T2-weighted magnetic resonance images.

From the research significance and status, it can be seen that the application of deep learning algorithms in medical image processing is of great significance. However, intelligent diagnosis of ovarian cancer focuses on the use of ultrasound images and machine learning models.

Therefore, we applied CNNs to PET/CT multimodal images of ovaries for intelligent diagnosis to segment and classify benign and malignant ovarian cancer. We proposed a segmentation model by fusing U-Net-MobileNetv3 with the KiteNet for lesion edge learning. Besides, we introduced lesion-based image-blending CarveMix and Dice-CE loss function, which significantly improved the segmentation performance. We then used ConvNeXt as the benchmark network and Mixup, a data augmentation technique used for image classification, to improve the classification performance. The main contributions of this paper were: (1) We proposed a computer-aided diagnosis model for ovarian tumors segmentation and classification based on PET/CT images; (2) We fused the KiteNet with U-Net-MobileNetv3 to extract more lesion edge features and introduced the CarveMix and Dice-CE loss to improve the results of segmentation; (3) We used ConvNeXt as benchmark network and Mixup for data augmentation to improve the classification performance; (4) Our models achieved high Dice and ACC for ovarian tumors segmentation and classification, which indicated the considerable significance of our models for the ovarian cancer diagnosis.

## Methods

### PET/CT ovarian tumor data collection

^18^F-FDG PET/CT imaging adopts the Biography mCT flow 64 PET/CT (Siemens Medical Solutions USA, Inc.). ^18^F-FDG (radiochemical pure > 95%) was provided by Shanghai Atomic Kexing Pharmaceutical Co., Ltd., China. All patients were subjected to at least 6 h of fasting before scanning, and the blood glucose level was reported below 11.1 mmol/L. All patients were injected with ^18^F-FDG 3.70 MBq/kg body mass through the superficial vein at the elbow, wait for the patient to lie down for 60 min, perform routine CT scan from the patient’s head to the mid femur, and then perform PET scan. As for CT scanning parameters, slice thickness was 3 mm under 170 mA and 120 kV voltage. The PET data was iteratively reconstructed by OSEM, and the attenuation correction of the PET images was carried out using the non-contrast CT transmission scan data.

**Fig. 1:**
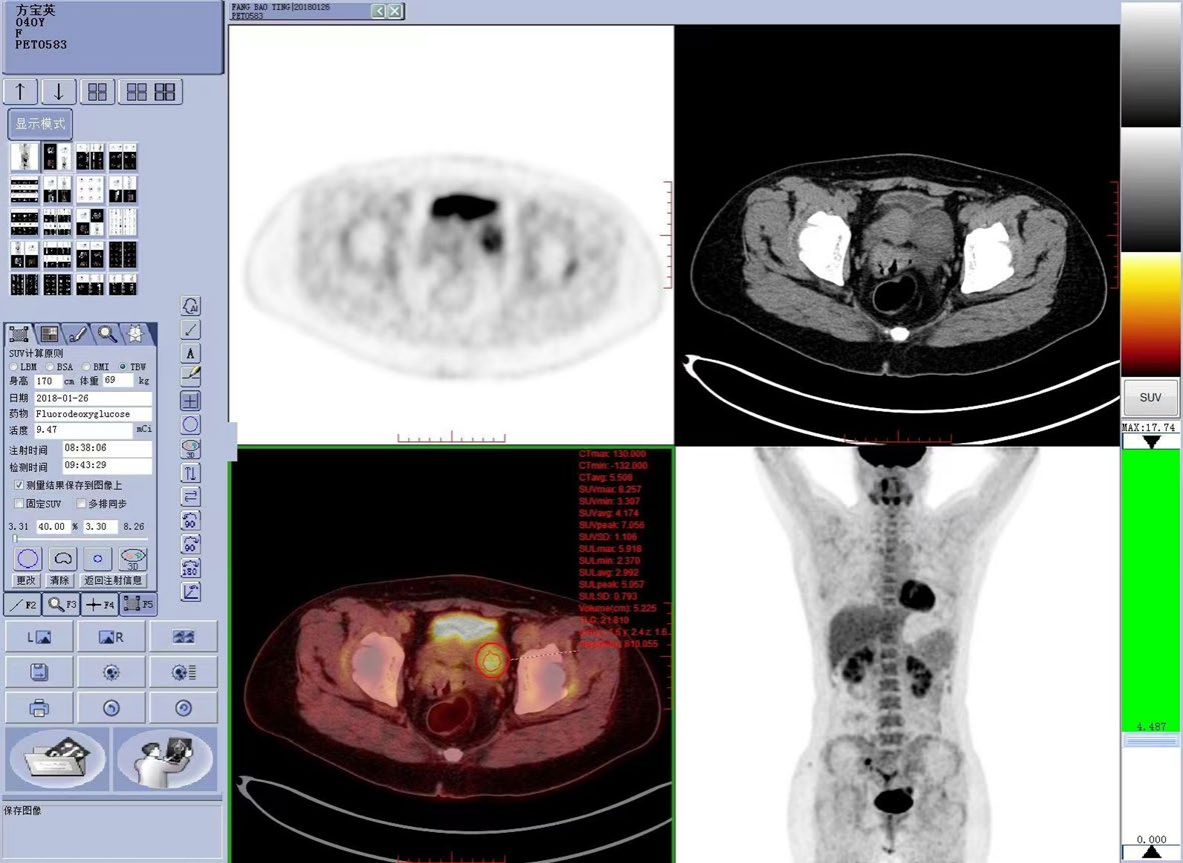
PET/CT working interface for intelligent diagnosis and treatment system (images and parameters).

The acquired whole-body ^18^F-FDG PET/CT images were transferred to the Siemens MMWP workstation, and the TrueD software performed image analysis and diagnosis in the cross-sectional, coronal, sagittal, and MIP planes, the working interface for intelligent diagnosis and treatment system was as below (Fig. 1). The lesion Region of interest (ROI) was delineated using the 3D threshold method on the MMWP workstation, and the workstation automatically delineated the volume of interest (VOI) using 40% of the maximum standard uptake value (SUVmax) as the threshold and obtained its SUVmax, metabolic tumor volume (MTV), and total lesion glycolysis (TLG). Draw a 10 mm diameter ROI at the aortic arch, measure the SUVmax of the mediastinal blood pool, and calculate the SUVmax ratio of the lesion to the mediastinal blood pool.

All masks were subsequently reviewed and manually corrected by two experienced nuclear medicine physicians (with 8 and 10 years of clinical experience, respectively) to ensure anatomical plausibility and alignment with CT morphology.

To assess inter-observer variability, we randomly selected 50 cases (≈ 10% of the dataset) and had both experts independently annotate the lesion masks. The inter-rater Dice coefficient was 0.91 ± 0.06, indicating high agreement. Discrepancies were resolved through consensus discussion. This validated subset was used as the final ground truth for model training and evaluation.

All image preprocessing was conducted using the Siemens MMWP workstation. The PET and CT image pairs were inherently aligned and co-registered at the scanner’s reconstruction station, ensuring precise spatial alignment between metabolic and anatomical information. To ensure data consistency across the dataset, all PET and CT images were resampled to a common isotropic voxel size for standardization. Regarding intensity normalization, CT Hounsfield Unit (HU) values were clipped to a range of − 150 to 250 HU to exclude air and bone extremes, while PET intensity values were normalized to correct for variability in injected dose and patient metabolism.

In our study, PET and CT images were processed as independent unimodal inputs, rather than being fused into a single multimodal representation. Specifically:

Each PET image (with its corresponding lesion mask) was used as an individual sample for both segmentation and classification tasks. Similarly, each CT image (with the same lesion mask derived from PET-based delineation) was also treated as a separate sample. No fusion strategy—neither early fusion (e.g., channel concatenation), feature-level fusion, nor late fusion—was applied in our current pipeline.

The dataset used in this experiment was the PET/CT image dataset of ovarian tumors provided by Zhejiang Provincial Hospital of Chinese Medicine. Our dataset consists exclusively of patients diagnosed with ovarian tumors confirmed by pathology; therefore, no healthy (non-tumor) cases are included. All subjects are female, consistent with the anatomical nature of ovarian cancer. All patients in this study were confirmed by pathological staining and immunohistochemistry. The images in this dataset have been labeled, including 773 images of benign tumors from 50 patients and 455 images of malignant tumors from 57 patients with ovarian tumors. The image size was 512 × 512. Each PET/CT image had a mask for the corresponding lesion area. Inclusion criteria: (1) Adequate signal-to-noise ratio; (2) Appropriate attenuation correction; (3) Correct reconstruction parameters; (4) No significant motion artifacts; (5) Adequate spatial resolution; (6) Accurate SUV quantification; (7) No obvious image contamination or artifacts; (8) Uptake of imaging agent (^18^F-FDG) in one or both ovaries; (9) Coverage extends from the patient’s head to the middle of the femur; (10) Scanning time points adhere to the protocol. Exclusion criteria: (1) Those who did not meet the above scanning protocol; (2) There was no uptake of imaging agent (^18^F-FDG) in both ovaries.

### Establishment of PET/CT classification model for ovarian tumors

In preliminary comparative experiments, EfficientNet^22^, Swin-Transformer^23^, DenseNet^24^, and ConvNeXt^25^ were used to determine the model with the best classification performance. We used Horizontal flip, Vertical flip and Random rotation as the basic data augmentation. In addition to those basic data augmentation used by CNNs, Mixup^26^ was used to increase the amount of training data. ACC was selected as the evaluation index for classifying ovarian tumor PET/CT images^27^.

### Establishment of PET/CT segmentation model for ovarian tumors

**Fig. 2:**
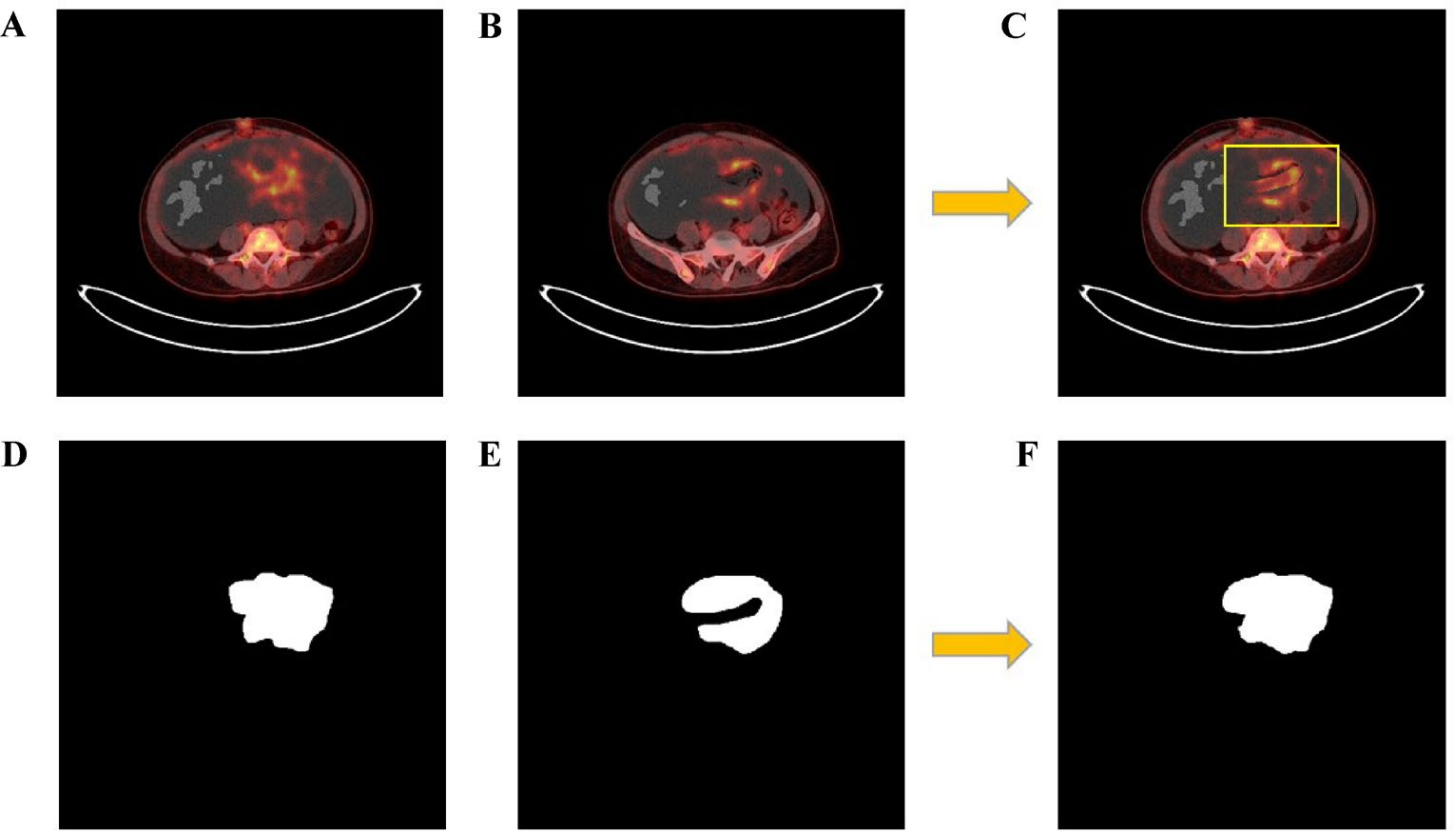
Schematic illustration of the CarveMix data augmentation strategy for segmentation. (**A**, **B**) Two original PET/CT images with their corresponding lesion masks (**D**, **E**). (**C**) A new synthetic image is generated by blending the foreground lesion region from image (**A**) into the background of image (**B**). (**F**) The corresponding synthetic mask is created by placing the lesion mask from (**D**) at the same location within the background of (**E**).

FCN^28^, Deeplabv3^29^, U-Net, U-Net-MobileNetv3, and U-Net-VGG were used for preliminary comparative experiments and to obtain the model with the best segmentation. The Horizontal flip, Vertical flip and Random rotation were used as the basic data augmentation. In addition to the basic data augmentation used by CNNs, CarveMix data augmentation method was introduced into the segmentation model^30^ (Fig. 2).

This lesion-aware augmentation increases data diversity while preserving anatomical plausibility, thereby improving model generalization.

#### A network for learning at the edge of the lesion

**Fig. 3:**
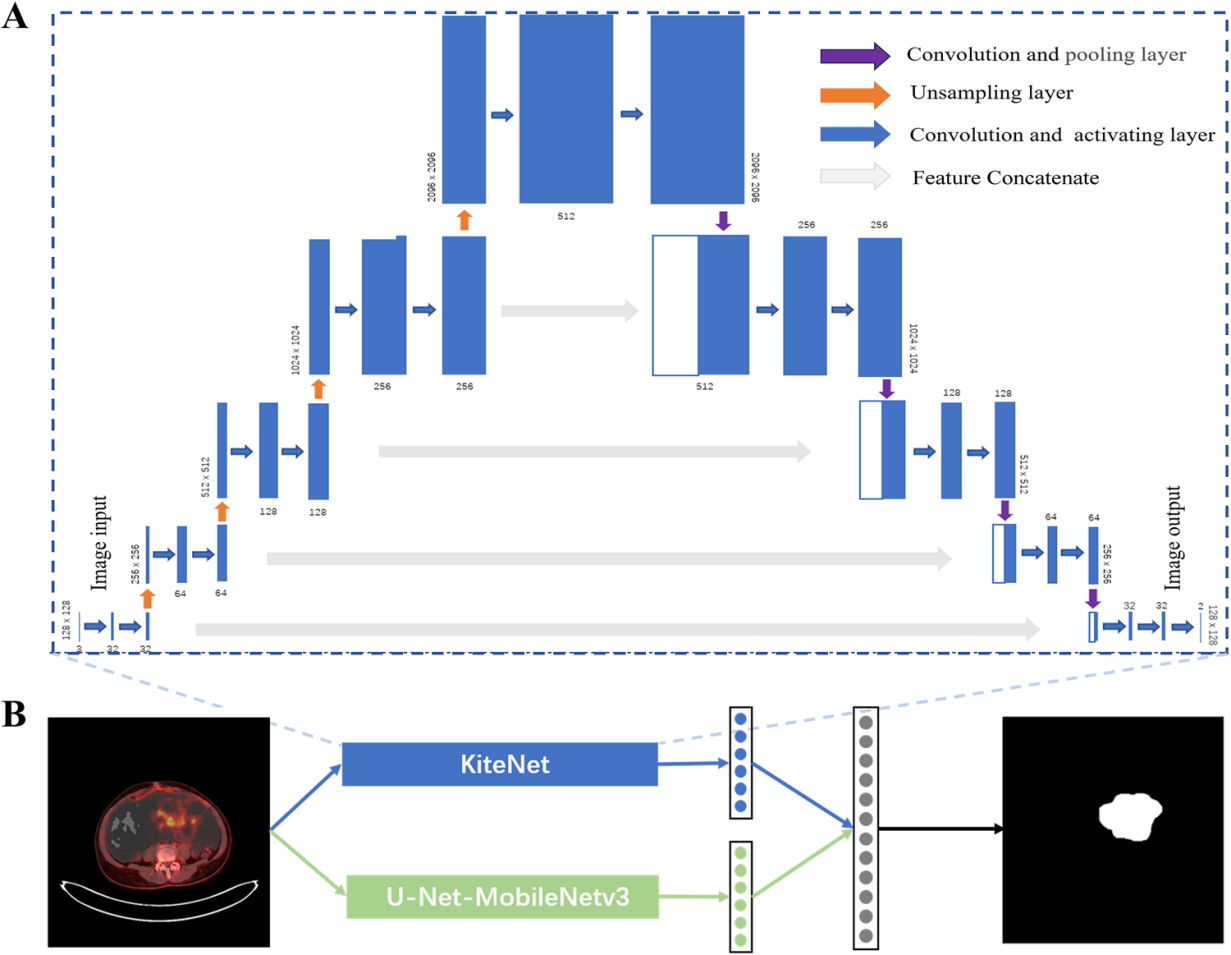
Schematic diagram of the performance improvement of the segmentation model. (**A**) Network structure diagram of KiteNet. (**B**) Schematic diagram of the fusion of KiteNet and U-Net-MobileNetv3 models.

Because the imaging quality of PET images was low, and the lesion boundary was difficult to accurately segment and recognize, KiteNet was introduced into the segmentation model (Fig. 3A). After this, the edge features of the lesion could be extracted. However, due to the design of the network structure, the receptive field of the encoder part was concentrated in a small part of the original lesion area, so the network could only learn fine features, and for important features such as shape and size, the limitation of the receptive field made it impossible to extract them. U-Net and its modifications have been remarkably successful in many medical image segmentation tasks. The integrated U-Net-MobileNetv3 expands the receptive field to encode global features (shape, size). Therefore, U-Net-MobileNetv3 was model-integrated with KiteNet (Fig. 3B). After the two networks extracted features from the original image, the two parts of the information were fused with features, and finally, the original image size was restored.

For the input image P, the encoding of U-Net-MobileNetv3 can be calculated as:

1 $$P_{i + 1}^{u} = E_{i}^{u} \left( {P_{i}^{u} } \right), \quad i = 1, 2, 3, 4.$$

Here, ${P}_{1}^{u}=P, {E}_{i}^{u}$ is the combination of the convolutional layer, activation layer and upsampling layer structure of the U-Net-MobileNetv3 encoder. The encoding of KiteNet can be calculated as:

2 $$P_{i + 1}^{k} = E_{i}^{k} \left( {P_{i}^{k} } \right),\quad i = 1, 2, 3, 4.$$

Here, ${P}_{1}^{k}=Down(P)$. Since KiteNet was upsampled in the encoder section, an excessively large input image size may cause an insufficient memory error. The PET/CT images were scaled to 128 × 128 and then entered into KiteNet. ${E}_{i}^{u}$ was the combination of the convolutional layer, activation layer and upsampling layer structure of the KiteNet encoder. Then, the decoder of models was below:

3 $$\begin{array}{*{20}l} {Q_{1}^{u} = D_{1}^{u} (P_{5}^{u} ),} \hfill & {Q_{1}^{k} = D_{1}^{k} (P_{5}^{k} )} \hfill \\ {Q_{1}^{u} ,P_{4}^{u} \mathop{\longrightarrow}\limits^{concat}R_{1}^{u} ,} \hfill & {Q_{1}^{k} ,P_{4}^{k} \mathop{\longrightarrow}\limits^{concat}R_{1}^{k} ,} \hfill \\ {Q_{2}^{u} = D_{2}^{u} (R_{1}^{u} ),} \hfill & {Q_{2}^{k} = D_{2}^{k} (R_{1}^{k} ).} \hfill \\ {Q_{2}^{u} ,P_{3}^{u} \mathop{\longrightarrow}\limits^{concat}R_{2}^{u} ,} \hfill & {Q_{2}^{k} ,P_{3}^{k} \mathop{\longrightarrow}\limits^{concat}R_{2}^{k} .} \hfill \\ {Q_{3}^{u} = D_{3}^{u} (R_{2}^{u} ),} \hfill & {Q_{3}^{k} = D_{3}^{k} (R_{2}^{k} ).} \hfill \\ {Q_{3}^{u} ,P_{2}^{u} \mathop{\longrightarrow}\limits^{concat}R_{3}^{u} ,} \hfill & {Q_{3}^{k} ,P_{2}^{k} \mathop{\longrightarrow}\limits^{concat}R_{3}^{k} .} \hfill \\ {Q_{4}^{u} = D_{4}^{u} (R_{2}^{u} ),} \hfill & {Q_{4}^{k} = D_{4}^{k} (R_{2}^{k} ).} \hfill \\ {Q_{4}^{u} ,P_{1}^{u} \mathop{\longrightarrow}\limits^{concat}R_{4}^{u} ,} \hfill & {Q_{4}^{k} ,P_{1}^{k} \mathop{\longrightarrow}\limits^{concat}R_{4}^{k} .} \hfill \\ {Q_{5}^{u} = D_{5}^{u} (R_{4}^{u} ),} \hfill & {Q_{5}^{k} = D_{5}^{k} (R_{4}^{k} ).} \hfill \\ \end{array}$$

Here, ${D}_{i}^{u}$ was the combination of the convolutional layer, activation layer and up sampling layer structure of the U-Net-MobileNetv3 decoder, ${D}_{i}^{k}$ was the combination of the convolutional layer, activation layer and pooling layer structure of the KiteNet decoder.

Finally, we resized features to be consistent and concatenate features from two networks to obtain the final segmentation result as follows:

4 $$\begin{gathered} Q_{6}^{k} = Up\left( {Q_{5}^{k} } \right). \hfill \\ Q = Concat\left( {Q_{6}^{k} ,Q_{5}^{u} } \right). \hfill \\ P^{seg} = Resize\left( Q \right). \hfill \\ \end{gathered}$$

#### Loss function

Cross-entropy loss and the Dice loss were combined to generate the Dice-CE loss function, which was more suitable for segmentation network^31^. The Cross-entropy loss focused on the pixel which might be influenced by the background of ovarian images while the Dice loss focused on the region of interest which was the tumor itself. The Dice-CE loss balanced the foreground and background of images which was designed as below:

5 $$\begin{aligned} L^{seg} = & \,L_{Dice - CE} = \alpha L_{CE}^{seg} + (1 - \alpha )L_{Dice} \\ = & \, - \frac{\alpha }{C}\sum\limits_{c = 1}^{C} {\left( {\sum\limits_{i = 1}^{N} {\sum\limits_{k = 1}^{2} {(r_{c}^{i,k} \log (s_{c}^{i,k} ))} } } \right)} \\ & \, + \frac{(1 - \alpha )}{C}\sum\limits_{c = 1}^{C} {\left( {1 - 2\frac{{\sum\nolimits_{i = 1}^{N} {\sum\nolimits_{k = 1}^{2} {(r_{c}^{i,k} s_{c}^{i,k} ) + \varepsilon } } }}{{\sum\nolimits_{i = 1}^{N} {\sum\nolimits_{k = 1}^{2} {(r_{c}^{i,k} + s_{c}^{i,k} )} } + \varepsilon }}} \right)} \\ \end{aligned}$$

r_c_^i,k^ and s_c_^i,k^ represented the true and prediction label of pixel I; N was the total number of pixels; C was the total number of images entered into the neural network per batch; the subscript c represented the c th image; α was the weight of the loss function, we chose α = 0.5; ε was to avoid regular terms with a denominator that was too small. The Dice coefficient is the segmentation model evaluation index.

### Experience settings

This study was developed using the PyTorch deep learning framework, version 1.7.0. The CPU in the hardware was an Intel i5.4460, the GPU was an NVIDIA GeForce GTX 1080ti, and all experiments were implemented in Python 3.6. A batch size of 16 and AdamW optimizer with a learning rate of 10^–4^ were used for all models. We trained models for 40 epochs using a 0.1 decay learning rate scheduler with 10 epochs. Fivefold cross-validation was used to evaluate the performance of ovarian tumor segmentation and classification models. Regarding the five-fold cross-validation, patient-level splitting was performed to avoid data leakage (i.e., all images from the same patient appear only in one fold). The distribution of patients and images across the five folds is summarized in the Table 1.

**Table 1:** Distribution of patients and PET/CT images across the five-fold cross-validation.

Fold number	Benign patients	Malignant patients	Benign images	Malignant images
1	10	11	155	91
2	10	12	154	92
3	10	11	156	89
4	10	12	153	93
5	10	11	155	90
Total	50	57	773	455

## Result

### Performance of the proposed model over baseline architectures

**Fig. 4:**
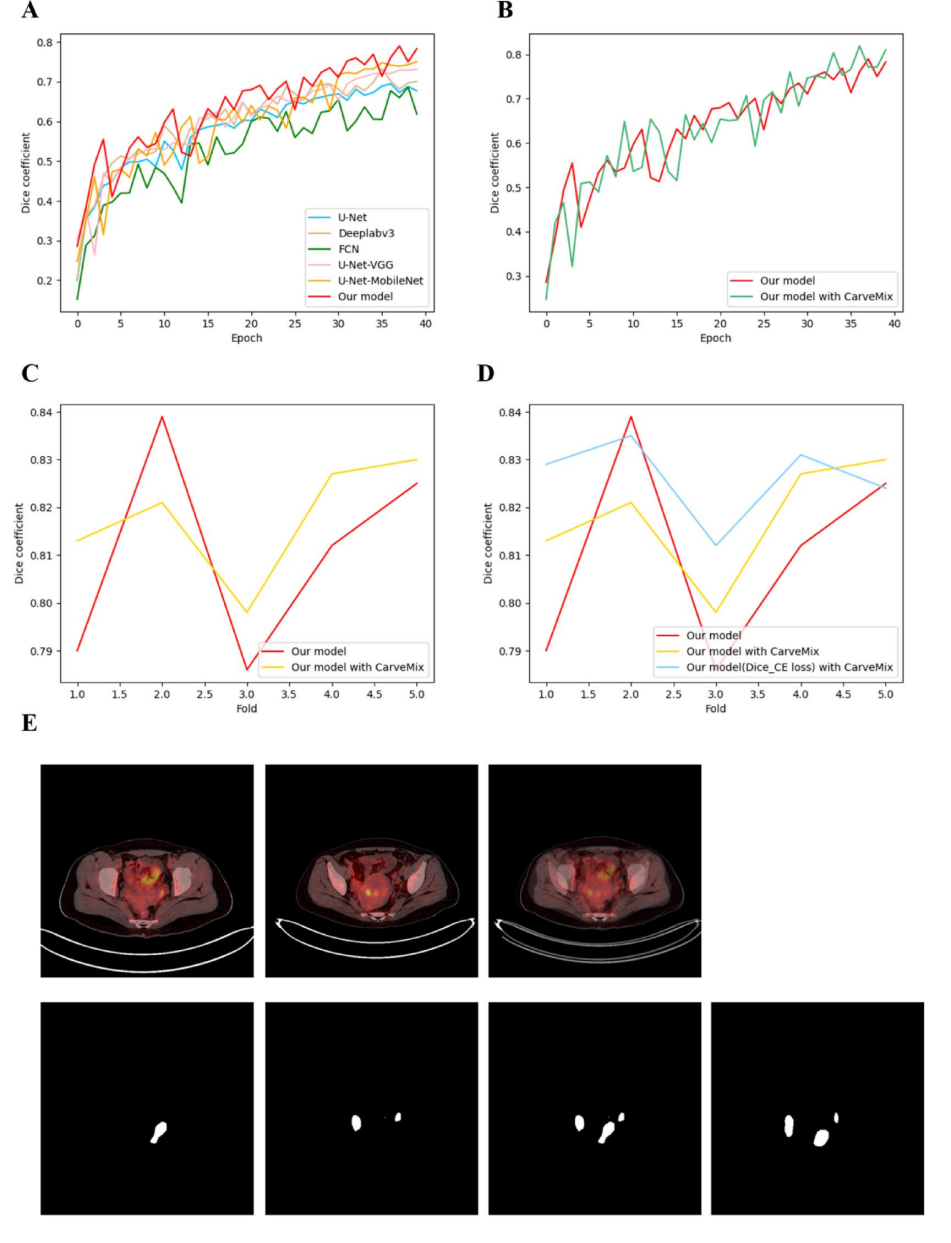
Quantitative and qualitative evaluation of ovarian tumor segmentation performance. (**A**) Dice coefficient vs. training epoch for various baseline models on the first fold of cross-validation. All models converge after ~ 30 epochs; (**B**) Training curves of our proposed model with and without CarveMix augmentation, showing improved stability and peak performance with CarveMix; (**C**) Comparison of final Dice scores across all five folds: our model with CarveMix consistently outperforms the version without augmentation; (**D**) Ablation study on the loss function: using Dice-CE loss yields higher Dice than cross-entropy alone across all folds; (**E**) Qualitative segmentation results: from left to right—original PET image, ground-truth mask, segmentation by U-Net-MobileNetv3, by KiteNet, and by our proposed model. Our method achieves the closest alignment with the ground truth, especially at lesion boundaries.

It can be seen from the five-fold cross-validation results that our proposed segmentation model consistently outperforms all baseline architectures across multiple evaluation metrics (Table 2). Specifically, our model achieves a mean Dice coefficient of 0.811, IoU of 0.681, and Average Hausdorff Distance (AHD) of 5.4 pixels. In comparison, the best-performing baseline, U-Net-MobileNetv3, yields a Dice of 0.773, IoU of 0.630, and AHD of 9.2 pixels. This represents an absolute improvement of + 0.038 in Dice, + 0.051 in IoU, and a significant reduction of − 3.8 pixels in AHD. The other baselines—FCN (Dice: 0.634, AHD: 19.3 px), Deeplabv3 (Dice: 0.744, AHD: 10.9 px), U-Net (Dice: 0.667, AHD: 16.6 px), and U-Net-VGG (Dice: 0.747, AHD: 10.9 px)—also demonstrate inferior performance in both region overlap (Dice/IoU) and boundary accuracy (AHD). These results confirm that the integration of edge-aware features, lesion-based augmentation, and optimized loss function in our model leads to more accurate and geometrically faithful segmentation of ovarian tumors in PET/CT imaging. The relationship between the Dice coefficient and the number of network training epochs was demonstrated on the first PET/CT training and test set of ovarian tumors in the fivefold crossover experiment, which showed that the results of each model tended to stabilize after 30 epochs (Fig. 4A). Next, CarveMix was applied to our segmentation model and the performance and effectiveness of the segmentation model was further improved (Fig. 4B, C, E). The CarveMix increased the dataset size which provided more potential features of data and reduced the overfitting during the training. As the volume of data increased, the entire network’s performance gradually improved (Table 3). Furthermore, to verify the model’s robustness against inter-class variations, we conducted a post-hoc sensitivity analysis. The results showed no significant difference in segmentation performance between benign (Dice = 0.828 ± 0.011) and malignant (Dice = 0.823 ± 0.013) cases (*p* = 0.42), confirming that the model maintains consistent performance across different tumor types.

**Table 2:** Performance of FCN, Deeplabv3, U-Net, U-Net-MobileNetv3, and U-Net-VGG models for PET/CT image segmentation.

Fold	Model	Dice	IoU	AHD (px)
1	FCN	0.642	0.472	18.9
	Deeplabv3	0.721	0.564	12.7
	U-Net	0.677	0.511	16.2
	U-Net-VGG	0.731	0.576	12
	U-Net-MobileNetv3	0.75	0.6	10.8
	Segmentation model	0.79	0.653	6.2
2	FCN	0.632	0.462	19.4
	Deeplabv3	0.745	0.594	10.9
	U-Net	0.691	0.527	15.3
	U-Net-VGG	0.761	0.614	10.1
	U-Net-MobileNetv3	0.774	0.632	9.2
	Segmentation model	0.812	0.683	5.1
3	FCN	0.655	0.487	18.2
	Deeplabv3	0.752	0.602	10.4
	U-Net	0.672	0.505	16.5
	U-Net-VGG	0.742	0.59	11.3
	U-Net-MobileNetv3	0.789	0.651	8.3
	Segmentation model	0.839	0.721	4.3
4	FCN	0.629	0.459	19.6
	Deeplabv3	0.756	0.608	10.1
	U-Net	0.658	0.489	17.1
	U-Net-VGG	0.755	0.607	10.2
	U-Net-MobileNetv3	0.747	0.597	11
	Segmentation model	0.786	0.647	6.4
5	FCN	0.614	0.443	20.3
	Deeplabv3	0.748	0.598	10.7
	U-Net	0.636	0.465	18
	U-Net-VGG	0.75	0.6	10.8
	U-Net-MobileNetv3	0.803	0.67	7.5
	Segmentation model	0.825	0.703	4.8
Mean	FCN	0.634	0.465	19.3
	Deeplabv3	0.744	0.593	10.9
	U-Net	0.667	0.499	16.6
	U-Net-VGG	0.747	0.597	10.9
	U-Net-MobileNetv3	0.773	0.63	9.2
	Segmentation model	0.811	0.681	5.4
SD	–	0.023	0.028	0.8

The results were presented as the mean ± STD. *N* = 5/per group.

**Table 3:** Performance of different amounts of PET/CT image data for ovarian tumors.

Fold	Without CarveMix	M + M	M + 1.5 M	M + 2 M
	Dice/IoU/AHD	Dice/IoU/AHD	Dice/IoU/AHD	Dice/IoU/AHD
1	0.790/0.653/6.2	0.805/0.670/5.3	0.806/0.672/5.2	0.813/0.681/4.8
2	0.812/0.683/5.1	0.809/0.677/5.5	0.817/0.689/4.9	0.821/0.695/4.6
3	0.839/0.721/4.3	0.790/0.653/6.2	0.798/0.664/5.8	0.798/0.664/5.8
4	0.786/0.647/6.4	0.821/0.695/4.6	0.823/0.698/4.5	0.827/0.703/4.3
5	0.825/0.703/4.8	0.810/0.678/5.4	0.804/0.670/5.3	0.830/0.707/4.2
Mean	0.811/0.681/5.4	0.807/0.675/5.4	0.810/0.679/5.1	0.818/0.698/4.7
SD	–/0.028/0.8	–/0.027/0.8	–/0.026/0.7	–/0.025/0.7

The results were presented as the mean. *N* = 5/per group. M was the original dataset without CarveMix data augmentation.

Finally, we incorporated the Dice-CE loss function into our segmentation model. As shown in Fig. 4D and Table 4, replacing the standard cross-entropy loss with Dice-CE further improved performance: the Dice coefficient increased from 0.818 to 0.826 (+ 0.008), the IoU rose from 0.690 to 0.703 (+ 0.013), and the Average Hausdorff Distance (AHD) decreased from 4.7 to 4.3 pixels (− 0.4 px). These results demonstrate that the Dice-CE loss effectively balances foreground–background learning and enhances both region overlap and boundary accuracy.

**Table 4:** Performance of the model, model with CarveMix, model with CarveMix after introducing the Dice-CE loss function.

Fold	Model			Model with C			Model with C(DCEL)		
	Dice	IoU	AHD (px)	Dice	IoU	AHD (px)	Dice	IoU	AHD (px)
1	0.79	0.653	6.2	0.813	0.681	4.8	0.829	0.707	4
2	0.812	0.683	5.1	0.821	0.695	4.6	0.835	0.721	3.8
3	0.839	0.721	4.3	0.798	0.664	5.8	0.812	0.683	5.1
4	0.786	0.647	6.4	0.827	0.703	4.3	0.831	0.706	4.2
5	0.825	0.703	4.8	0.83	0.707	4.2	0.824	0.7	4.4
Mean	0.811	0.681	5.4	0.818	0.69	4.7	0.826	0.703	4.3
SD	0.023	0.028	0.8	0.019	0.024	0.7	0.012	0.017	0.6

The results were presented as the mean. *N* = 5 per group.

Model: our segmentation model, model with C: the segmentation model after adding CarveMix, model with C (DCEL): the segmentation model using the CarveMix and Dice-CE loss functions.

### Mixup improved the ACC and AUC of classification model for ovarian tumors

**Fig. 5:**
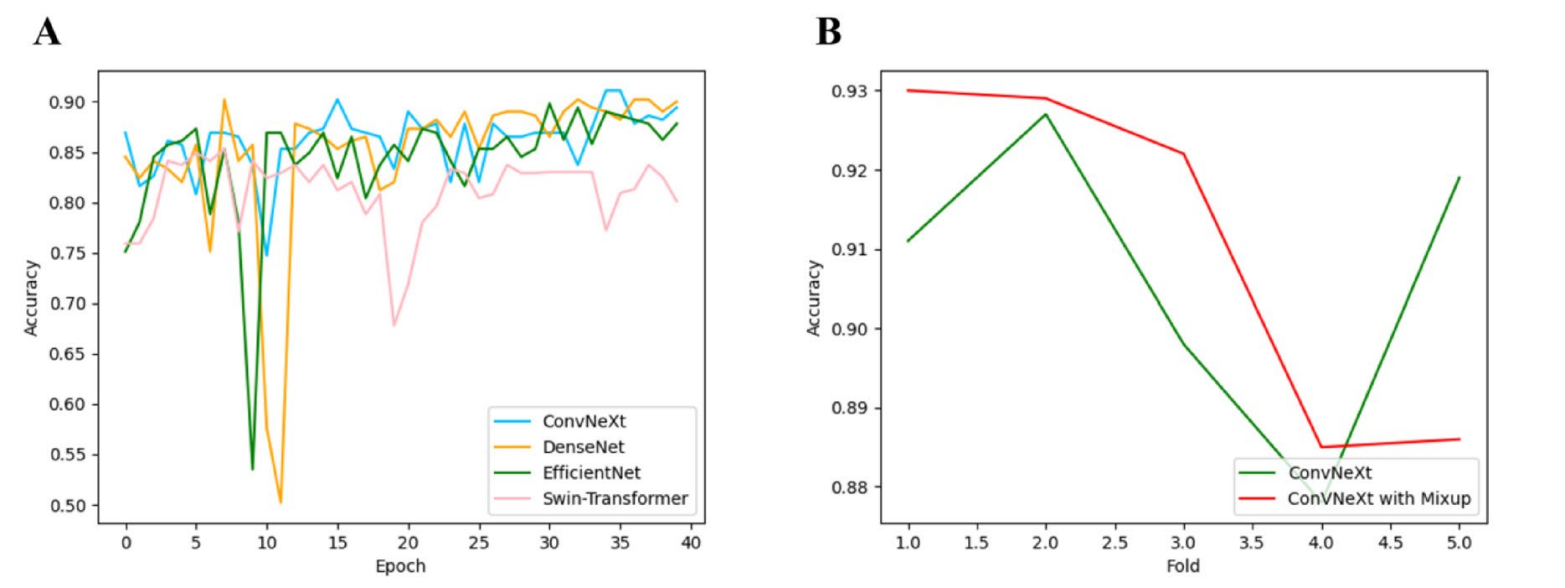
Classification comparison of ovarian tumors in PET/CT images. (**A**) The relationship between the ACC rate on the training set and the test set of the first ovarian tumors in PET/CT data and the number of network training epochs; (**B**) The ACC of our model with/without Mixup in the fivefold crossover experiment; Fold (0,5) represents the fivefold cross experiment, and the ordinate was the ACC.

PET/CT images of ovarian tumors were classified using four baseline deep learning models: DenseNet, EfficientNet, ConvNeXt, and Swin-Transformer. As shown in Fig. 5A and Table 5, their performance varied across folds. The mean accuracy (ACC) and area under the ROC curve (AUC) were as follows:

- DenseNet: ACC = 0.898 ± 0.014, AUC = 0.923 ± 0.013
- EfficientNet: ACC = 0.895 ± 0.011, AUC = 0.920 ± 0.012
- ConvNeXt: ACC = 0.907 ± 0.019, AUC = 0.938 ± 0.015
- Swin-Transformer: ACC = 0.848 ± 0.016, AUC = 0.874 ± 0.014

**Table 5:** The performance of DenseNet, EfficientNet, ConvNeXt, and Swin-transformer models for ovarian tumors PET/CT image classification.

Fold	ConvNeXt (ACC/AUC)	DenseNet (ACC/AUC)	EfficientNet (ACC/AUC)	Swin-transformer (ACC/AUC)
1	0.911/0.938	0.902/0.927	0.898/0.922	0.837/0.862
2	0.927/0.951	0.914/0.936	0.910/0.933	0.825/0.850
3	0.898/0.923	0.907/0.929	0.882/0.908	0.856/0.881
4	0.878/0.905	0.890/0.912	0.886/0.910	0.864/0.889
5	0.919/0.945	0.878/0.903	0.898/0.924	0.858/0.885
Mean	0.907/0.938	0.898/0.923	0.895/0.920	0.848/0.874
SD	0.019/0.015	0.014/0.013	0.011/0.012	0.016/0.014

The results were presented as the mean ± SD. *N* = 5 per group.

ConvNeXt achieved the highest performance in both metrics, confirming its suitability as the backbone for our classification pipeline. Notably, the relatively high AUC values (all > 0.87) indicate robust discriminative ability despite mild class imbalance, while the lower AUC–ACC gap for ConvNeXt (Δ = 0.031) suggests better calibration and reduced bias toward the majority class compared to other models. As was shown in Fig. 5A, the results of each classification model tended to stabilize after 25 epochs. Since the average ACC and best ACC of ConvNeXt were highest, and the fluctuation was smaller than that of DenseNet, which had excellent performance, ConvNeXt was selected as the benchmark network for the classification of benign and malignant ovarian tumors. Next, Mixup was applied to our classification model, and the performance and effect of the classification model have been further improved (Fig. 5B). The Mixup augmentation expanded the effective training dataset size, thereby enriching feature diversity and mitigating overfitting during model training. As the augmentation intensity increased (from M to M + 2 M), both classification accuracy (ACC) and discriminative ability (AUC) improved progressively: ACC rose from 0.907 to 0.912, while AUC increased from 0.938 to 0.947 (Table 6). This consistent improvement across complementary metrics confirms that Mixup not only enhances generalization but also strengthens the model’s confidence calibration—particularly valuable in the presence of mild class imbalance.

**Table 6:** The performance of different amounts of PET/CT image data for ovarian tumors.

Fold	Without Mixup ACC/AUC	M + M ACC/AUC	M + 1.5 M ACC/AUC	M + 2 M ACC/AUC
1	0.911/0.938	0.923/0.949	0.928/0.954	0.930/0.956
2	0.927/0.951	0.915/0.940	0.918/0.943	0.929/0.954
3	0.898/0.923	0.907/0.932	0.911/0.936	0.922/0.947
4	0.878/0.905	0.881/0.908	0.863/0.889	0.885/0.910
5	0.919/0.945	0.903/0.928	0.894/0.919	0.896/0.921
Mean	0.907/0.938	0.906/0.937	0.903/0.932	0.912/0.947
SD	–/0.015	–/0.014	–/0.016	–/0.015

The results were presented as the mean. *N* = 5 per group. M was the original dataset without Mixup data augmentation. “M + kM”: the model trained on the original M samples plus kM synthetic samples generated by Mixup augmentation.

### Our segmentation model compared with U-Net-MobileNetv3 and KiteNet

**Fig. 6:**
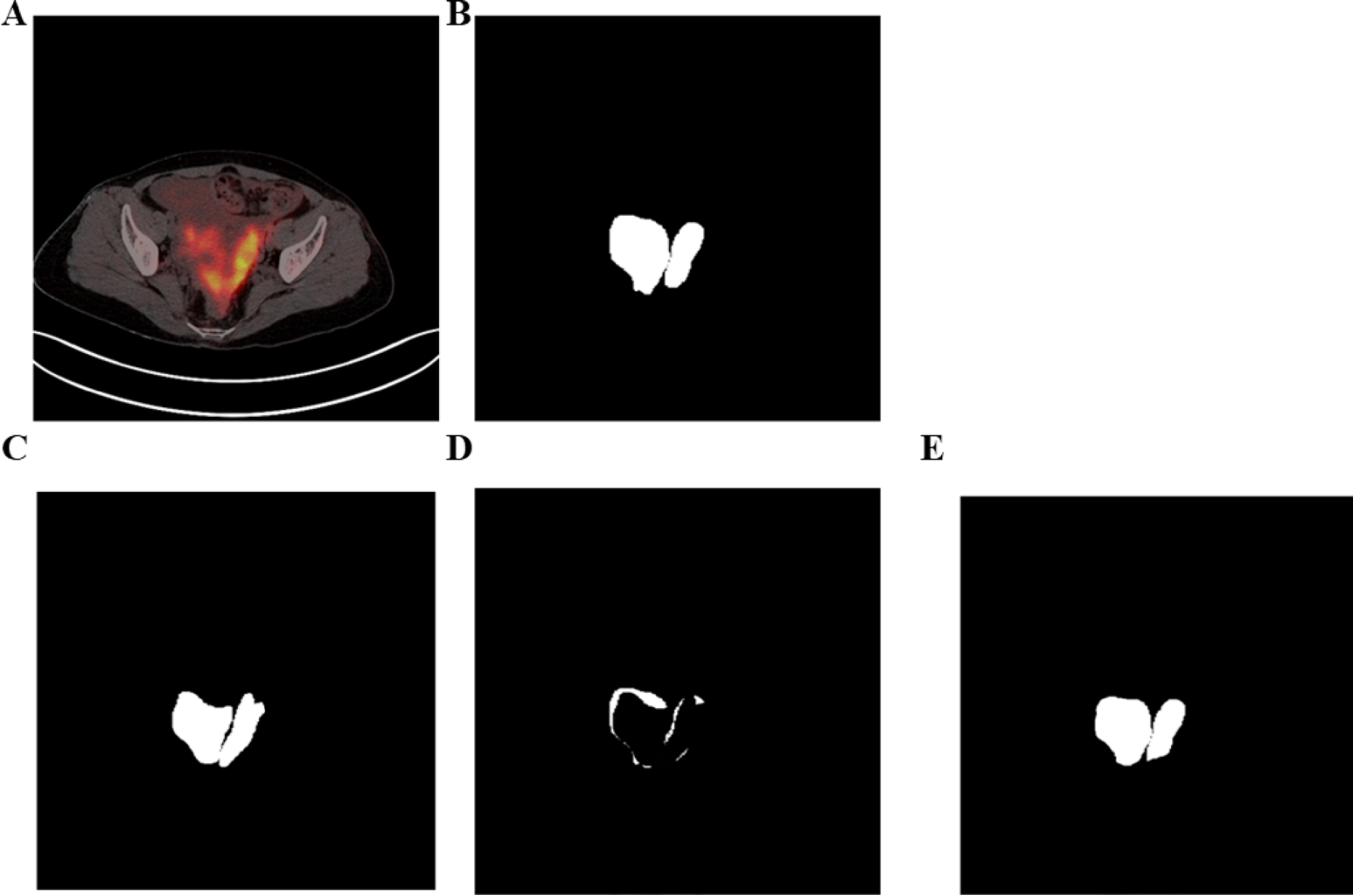
Actual effect of ovarian tumor segmentation. (**A**) was the original image, and (**B**) was the mask. (**C**) was the ovarian tumor segmentation effect of U-Net-MobileNetv3, (**D**) showed the ovarian tumor segmentation effect of KiteNet, and (**E**) was ovarian tumor segmentation effect of our model.

U-Net-MobileNetv3 and KiteNet were used to compare the effects of ovarian tumor segmentation with our segmentation model. Our model had a better segmentation effect than U-Net-MobileNetv3 and KiteNet (Fig. 6). The segmentation performance was evaluated across three models using multiple metrics (Table 7). KiteNet achieved a mean Dice coefficient of 0.444, corresponding to an IoU of 0.305 and an Average Hausdorff Distance (AHD) of 19.3 pixels, indicating limited accuracy in both region overlap and boundary delineation. U-Net-MobileNetv3 showed substantially improved performance with a Dice of 0.773, IoU of 0.630, and AHD of 9.2 pixels. In contrast, our proposed segmentation model attained the highest scores: Dice = 0.826, IoU = 0.694, and AHD = 4.3 pixels, demonstrating superior lesion coverage and geometric fidelity. The significant reduction in AHD (− 4.9 px vs. U-Net-MobileNetv3) highlights the effectiveness of our edge-aware architecture and optimized training strategy.

**Table 7:** The performance of KiteNet, U-Net-MobileNetv3, and our model for PET/CT image segmentation of ovarian tumors in the fivefold crossover experiment.

Model	Fold	Dice	IoU	AHD (px)
KiteNet	1	0.473	0.319	18.6
	2	0.426	0.298	19.9
	3	0.442	0.311	19.1
	4	0.416	0.292	20
	5	0.461	0.314	18.7
	Mean	0.444	0.305	19.3
U-Net-MobileNetv3	1	0.75	0.6	10.8
	2	0.774	0.632	9.2
	3	0.789	0.651	8.3
	4	0.747	0.597	11
	5	0.803	0.67	7.5
	Mean	0.773	0.63	9.2
Segmentation model	1	0.829	0.707	4
	2	0.835	0.721	3.8
	3	0.812	0.683	5.1
	4	0.831	0.706	4.2
	5	0.824	0.703	4.4
	Mean	0.826	0.694	4.3

The results were presented as the mean. *N* = 5 per group.

## Discussion

PET/CT images have been widely used to diagnose clinical diseases, and artificially interpreted imaging is inefficient and susceptible to clinician subjectivity. This study proposed a new method based on deep learning fusion PET/CT information for intelligent diagnosis of ovarian tumor classification and segmentation.

Our model achieved high Dice and ACC for ovarian tumors segmentation and classification on PET/CT datasets. EfficientNet, Swin-Transformer, DenseNet, and ConvNeXt were first used to perform comparative experiments on ovarian tumor PET/CT image classification, and FCN, Deeplabv3, U-Net, U-Net-MobileNetv3, and U-Net-VGG were tried for optimal model of ovarian tumor PET/CT image segmentation. The results showed that ordinary models did not perform well enough on our datasets. One of the reasons was that the datasets were small, and the underlying network model was not sufficiently trained due to a lack of data. In response to this problem, ConvNeXt was selected as the benchmark network for benign and malignant classification of ovarian tumors, which had the highest average ACC and smaller fluctuation than that of DenseNet. An improved data augmentation method, Mixup, was introduced to enrich the training dataset, increased the amount of data used for model training, and enabled the network to fully utilize its performance, which could mix images between different classes. The second reason was that the imaging quality of PET images was low, and the lesion characteristics were blurred and not obvious. The lesion area of ovarian cancer was small, which made it difficult to accurately segment and identify the lesion boundary. To this end, KiteNet was introduced, which was integrated into U-Net-MobileNetv3 in the underlying segmentation model. The method of fivefold cross-validation was used to evaluate the model performance, and our model outperformed U-Net-MobileNetv3, the best of the common network models. CarveMix was applied to our segmentation model, and the performance and effect of the segmentation model were further enhanced. Finally, the Dice-CE loss function was added to improve the performance of the segmentation model further.

The results of ovarian cancer segmentation using the KiteNet model alone were limited, achieving a mean Dice of 0.444 and AHD of 19.3 pixels. This is primarily because KiteNet’s design prioritizes local edge detection via small-kernel convolutions. Such an architecture concentrates the receptive field on small localized regions, making it effective for fine features but insufficient for extracting holistic structural features like the overall shape and size of the tumor. And it could be seen that KiteNet was mainly used for marginal segmentation of ovarian cancer lesions^32^. When KiteNet alone was used to segment ovarian cancer, KiteNet was too ineffective from the evaluation indicators. When U-Net-MobileNetv3 and KiteNet are combined for the ovarian cancer segmentation, the segmentation effect was better than the commonly used segmentation model, proving that our model fusion integration was effective. The combination of U-Net-MobileNetv3 and KiteNet ensured that both fine-grained edges features (from KiteNet) and holistic structural features (from U-Net-MobileNetv3) were extracted. While enlarging the depth or width of U-Net or KiteNet could theoretically improve performance, our experiments suggest that such efforts yield limited gains due to inherent structural constraints. Simply increasing KiteNet’s depth introduces structural redundancy without resolving its fundamental receptive field limitations. Similarly, larger U-Net models still face inherent downsampling trade-offs that restrict their ability to refine fine boundaries. Therefore, we emphasize that architectural fusion (KiteNet + U-Net-MobileNetv3) is a more effective strategy than mere scale expansion. Simply enlarging KiteNet’s architecture (e.g., adding layers) would not inherently resolve its fundamental constraint: the design prioritizes local edge detection via small-kernel convolutions. Expanding its depth could introduce redundancy without significantly improving global feature extraction (e.g., shape/size). Similarly, a larger U-Net-MobileNetv3 may still struggle with fine boundaries due to its inherent down sampling trade-offs. CarveMix is a special form of data augmentation for image segmentation^30^. Unlike image blending in classification, the lesion area information was required for CarveMix. A new image was obtained using CarveMix data augmentation.

The procedure is as follows:

- Two original PET/CT images (I_1_, I_2_) and their corresponding lesion masks (M_1_, M_2_) are randomly selected;
- The lesion region from I_1_ (defined by M_1_) is extracted;
- This lesion is directly pasted onto the background of I_2_ at the same anatomical location, producing a new synthetic image I_new_;
- The corresponding synthetic mask M_new_ is generated by placing M_1_ onto the background of M_2_.

Importantly, this is a hard replacement operation—not a weighted blend—so there is no “contribution weight” between components. The goal is to increase data diversity by creating novel lesion-background combinations while preserving anatomical plausibility.

Images obtained by segmentation using CarveMix alone do not necessarily have clinical or scientific significance. However, for the training of CNNs, the complexity of the images increased due to the mixing of the original image features, and the neural network could obtain a more powerful ability to extract features by learning the features of complex images, which allowed for adequate training of network performance and helped improve the ACC of the network and obtain more accurate segmentation effects. CNNs learned smaller-range and irregularly shaped lesion area features, which might be one of the reasons why CarveMix was effective^30,33^. In addition, CT and PET are both tomography and using CarveMix may also accidentally produce a composite image close to a certain layer’s real image.

For classification task, the model learned discriminative features between benign and malignant tumor images to generate final predictions. For segmentation task, the model primarily focused on distinguishing tumor regions from background tissue, with inter-class tumor variations having minimal impact on performance. Our sensitivity analysis further substantiates that inter-class tumor variations had minimal impact on the segmentation results. Despite the inherent data imbalance, the lack of statistical difference (*p* = 0.42) between benign and malignant segmentation scores suggests that the feature extraction capabilities of our fused architecture are equally effective for both tumor classes. This class imbalance may lead to model overfitting. To mitigate its impact, we employed data augmentation techniques such as CarveMix and Mixup to alleviate the issue to some extent. For segmentation, CarveMix was applied uniformly across all samples, regardless of class. Since segmentation focuses on lesion delineation (not class label), class imbalance is less critical. For classification, Mixup was applied randomly across all training samples without explicit minority-class oversampling. However, Mixup’s inherent property of interpolating between classes (including benign malignant pairs) helps regularize the decision boundary and mitigate bias toward the majority class.

We acknowledge that targeted oversampling of the malignant class (e.g., using SMOTE or class-balanced Mixup) could further improve robustness. This will be explored in future work.

These results confirm that our model maintains strong performance on the minority (malignant) class despite the imbalance. Importantly, the high AUC (0.947), sensitivity (0.892), and specificity (0.921) confirm that our model maintains strong discriminative performance on the minority (malignant) class despite the underlying data imbalance. In future work, we plan to explore class-balanced Mixup or focal loss to explicitly mitigate the impact of data imbalance.

To rigorously assess whether the performance gains of our proposed model are statistically significant, we performed paired two-tailed t-tests on the five-fold cross-validation results. Results of significance testing:

Segmentation: Our model (mean Dice = 0.826) vs. U-Net-MobileNetv3 (0.773): *p* = 0.008.

Classification: ConvNeXt With Mixup (mean ACC = 0.912) vs. ConvNeXt Without Mixup (0.907): *p* = 0.032.

Both comparisons yielded *p* < 0.05, confirming that the observed improvements are statistically significant.

Although the model performed best on the PET/CT dataset, its performance on other types of medical images, such as ultrasound or magnetic resonance images remains unknown, and further experiments could be carried out on the ultrasound and magnetic resonance datasets to optimize the network parameters. In the analysis of the results, KiteNet was confirmed as effective in learning the edge of the lesion, and the combination of U-Net and classification network can be considered in future research to integrate KiteNet with classification network to improve the network structure and optimize network performance. We can consider introducing multi-scale images to subdivide ovarian tumors, such as primary and secondary tumors, and malignant tumors in the malignant subclassification. Besides, we only evaluated on one dataset from a single institution, we can further incorporate additional dataset.

## Conclusions

This paper proposes a novel deep learning-based framework for intelligent diagnosis of ovarian tumors in PET/CT imaging, enabling both accurate lesion segmentation and reliable benign–malignant classification. The proposed segmentation model achieved a Dice coefficient of 0.826, an IoU of 0.694, and an AHD of 4.3 pixels, outperforming conventional models such as U-Net and Deeplabv3. Meanwhile, the classification model based on ConvNeXt with Mixup augmentation attained an accuracy of 0.912 and an AUC of 0.947, demonstrating superior diagnostic performance compared with existing approaches. These results indicate that the proposed method provides a robust and effective tool for assisting clinical decision-making in ovarian cancer diagnosis.

## Data Availability

The dataset used in this experiment was the PET/CT image dataset of ovarian tumors provided by Zhejiang Provincial Hospital of Chinese Medicine. The original code has been stored on github (https://github.com/XueruFan-user/petct.git). All data relevant to this study are available within the article. Further information could be obtained from the corresponding author (Prof. Lu Li), upon reasonable request.
